# Supplementary material for: Metallo-supramolecular branched polymer protects particles from air-water interface in single-particle cryo-electron microscopy
Source: Commun Biol. 2024 Jan 9;7:65. doi: 10.1038/s42003-023-05752-8 (PMC10776832; doi:10.1038/s42003-023-05752-8)
Supplement: Supplementary file 2 — Supplementary Information [file 42003_2023_5752_MOESM2_ESM.pdf]

Supplementary Information for

Metallo-Supramolecular Branched Polymer Protects Particles from Air-water Interface in Single-Particle Cryo-Electron Microscopy

Yixin Xu[1,*,$], Yuqi Qin[1,*], Lang Wang[2], Yingyi Zhang[3], Yufeng Wang[2,#], Shangyu Dang[1,4,5,#]

1Division of Life Science, The Hong Kong University of Science and Technology, Clear Water Bay, Hong Kong, China

[2]Department of Chemistry, The University of Hong Kong, Hong Kong, China

3Biological Cryo-EM Center, The Hong Kong University of Science and Technology, Clear Water Bay, Hong Kong, China

[4]Southern Marine Science and Engineering Guangdong Laboratory (Guangzhou), Guangzhou, China

[5]HKUST-Shenzhen Research Institute, Nanshan, Shenzhen 518057, China

[$]Present address: Department of Biology, Institute of Molecular Biology and Biophysics, ETH Zurich, Zurich, Switzerland

[*]These authors contributed equally.

[#]Correspondence: wanglab@hku.hk (Y.W.); sdang@ust.hk (S.D.).

This file includes:

Supplementary Figures 1 to 17

Supplementary Table 1

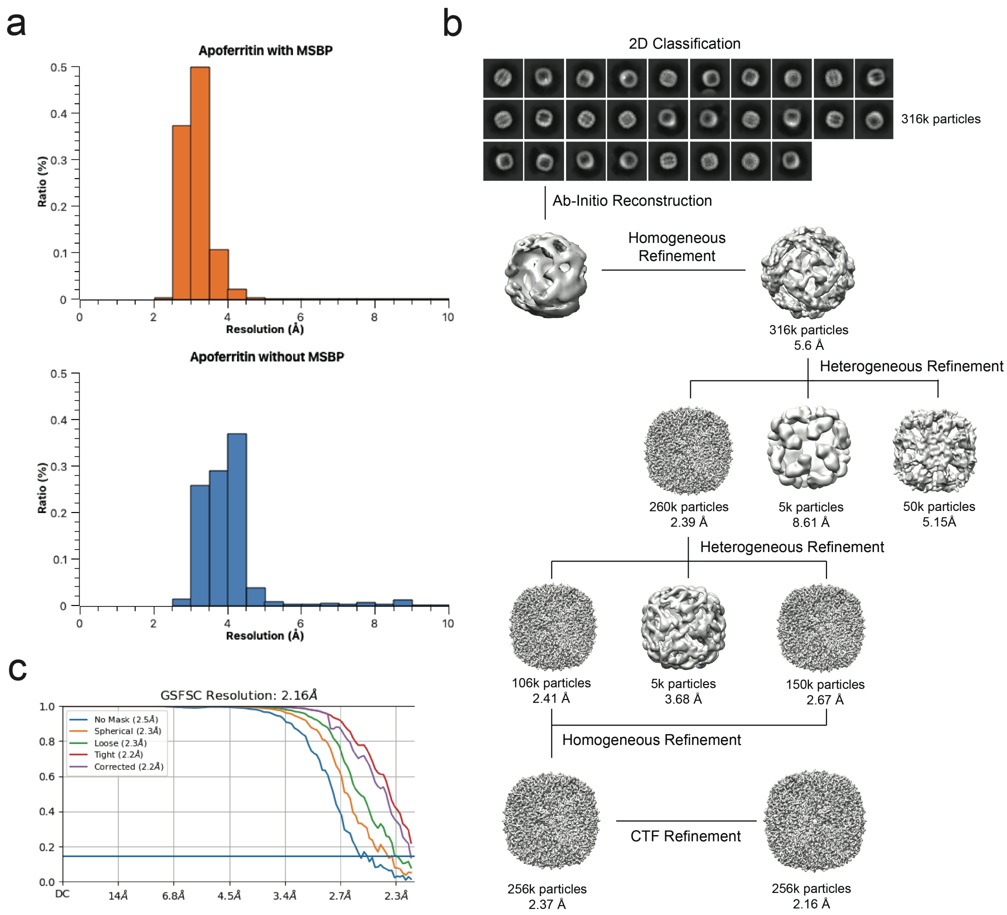

Supplementary Figure 1 Cryo-EM data processing of apoferritin with MSBP. (a) Statistics of estimated resolution of micrographs for apoferritin with or without MSBP. **(b)** Data processing workflow with number of particles and the reconstruction resolution indicated at every step. **(c)** FSC curves of the 3D reconstructions are also shown.

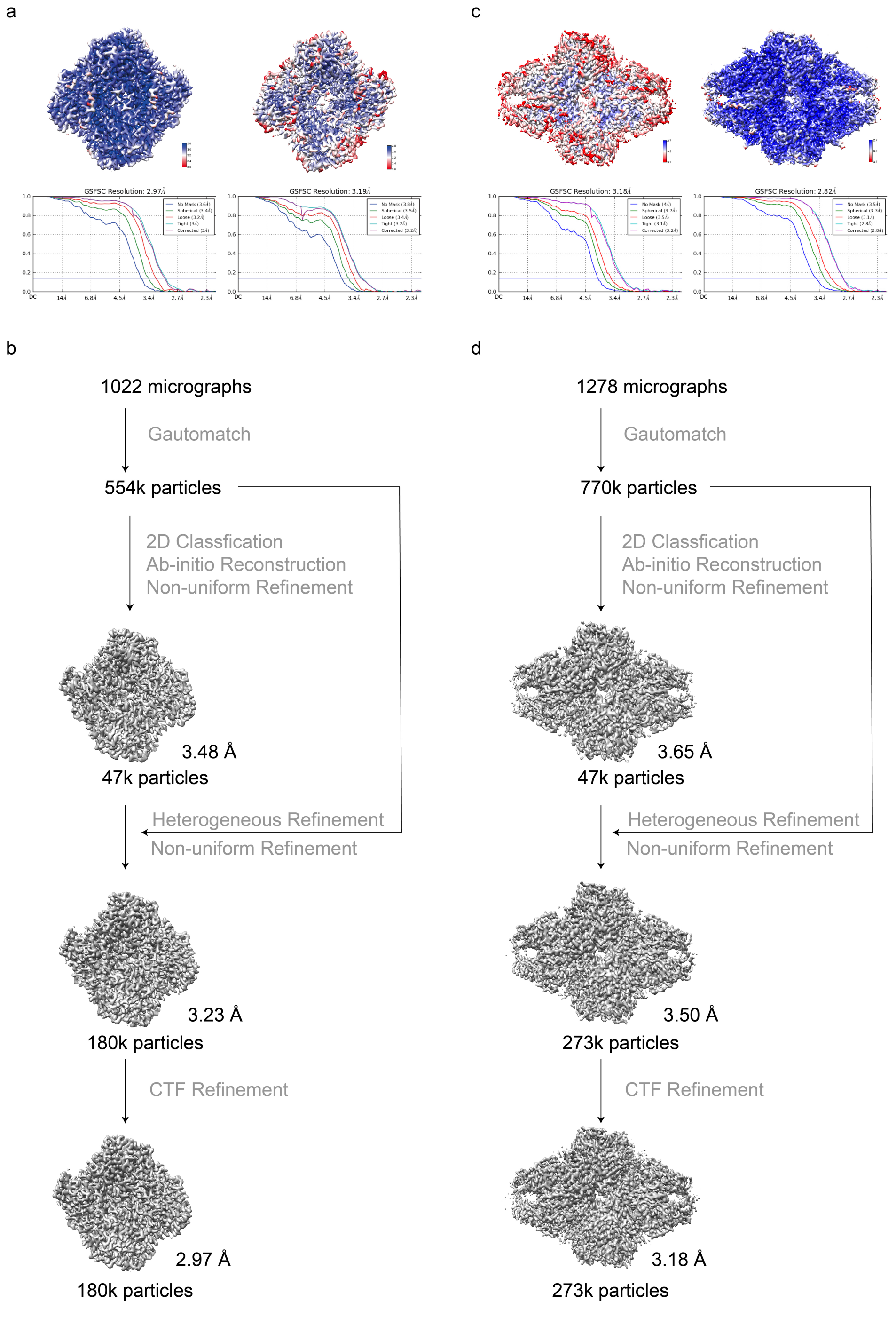

Supplementary Figure 2 Cryo-EM data processing of catalase and β-galactosidase. (a) Local resolution cryo-EM map and corresponding FSC curves of catalase with (left) or without (right) MSBP. **(b)** Data processing workflow of catalase with MSBP. The number of particles and the reconstruction resolution are indicated at every step. **(c)** Local resolution cryo-EM map and corresponding FSC curves of β-galactosidase with (left) or without (right) MSBP. **(d)** Data processing workflow of β-galactosidase with MSBP. The number of particles and the reconstruction resolution are indicated at every step.

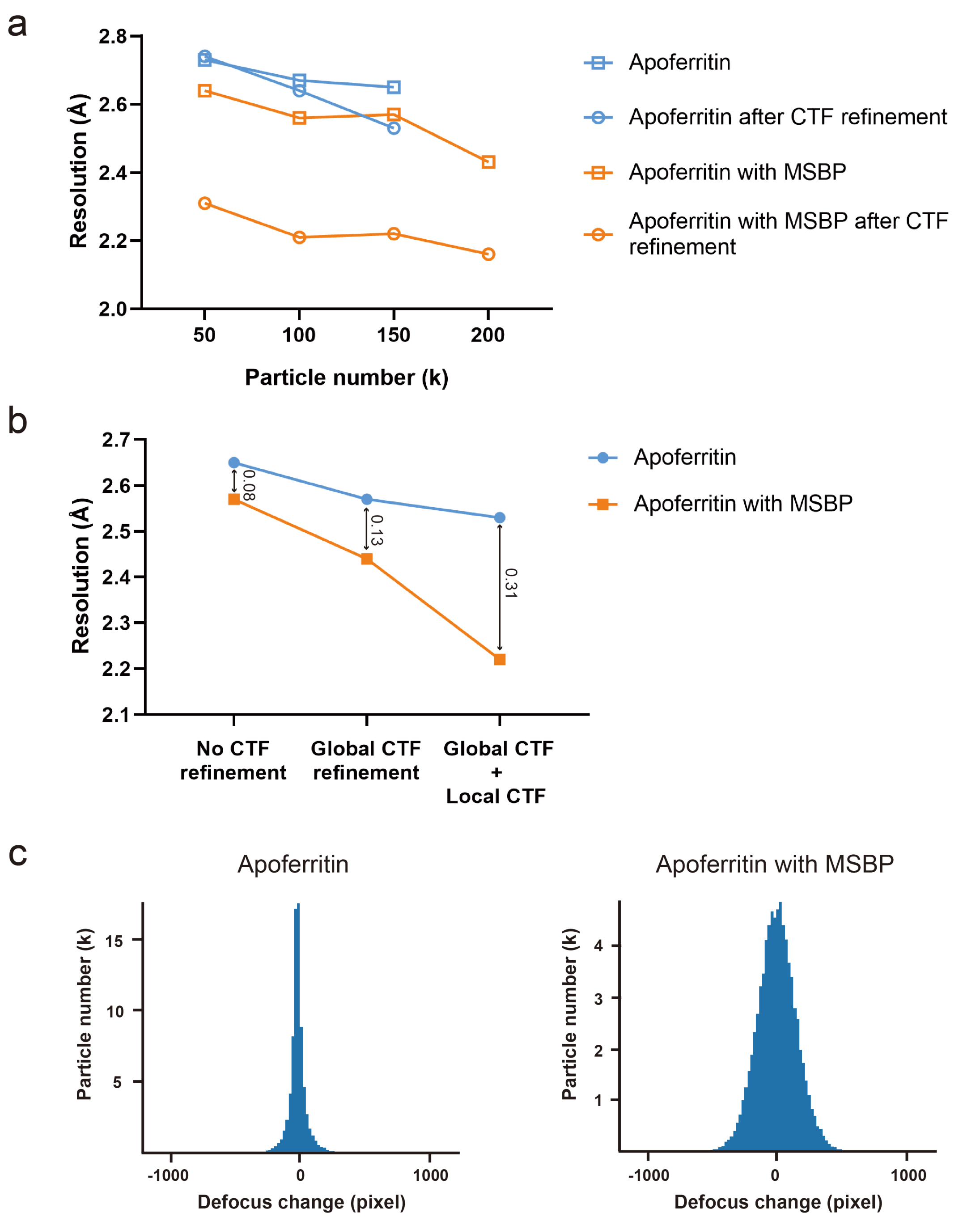

Supplementary Figure 3 Systematically comparison of resolution for apoferritin with or without MSBP. (a) The resolution corresponding to density map reconstructed with 50k, 100k, 150k and 200k particles is shown. Apoferritin with MSBP before (orange square) and after (orange circle) CTF refinement, apoferritin without MSBP before (blue square) and after (blue circle) ctf refinement are plotted for comparison. **(b)** Comparison of the resolution improvement by global and local CTF refinement with 150k particles of apoferritin (blue) and apoferritin with MSBP (orange). Resolution improvement of each strategy is indicated. **(c)** Particle distribution of defocus changes after local CTF refinement for apoferritin (left) or apoferritin with MSBP (right).

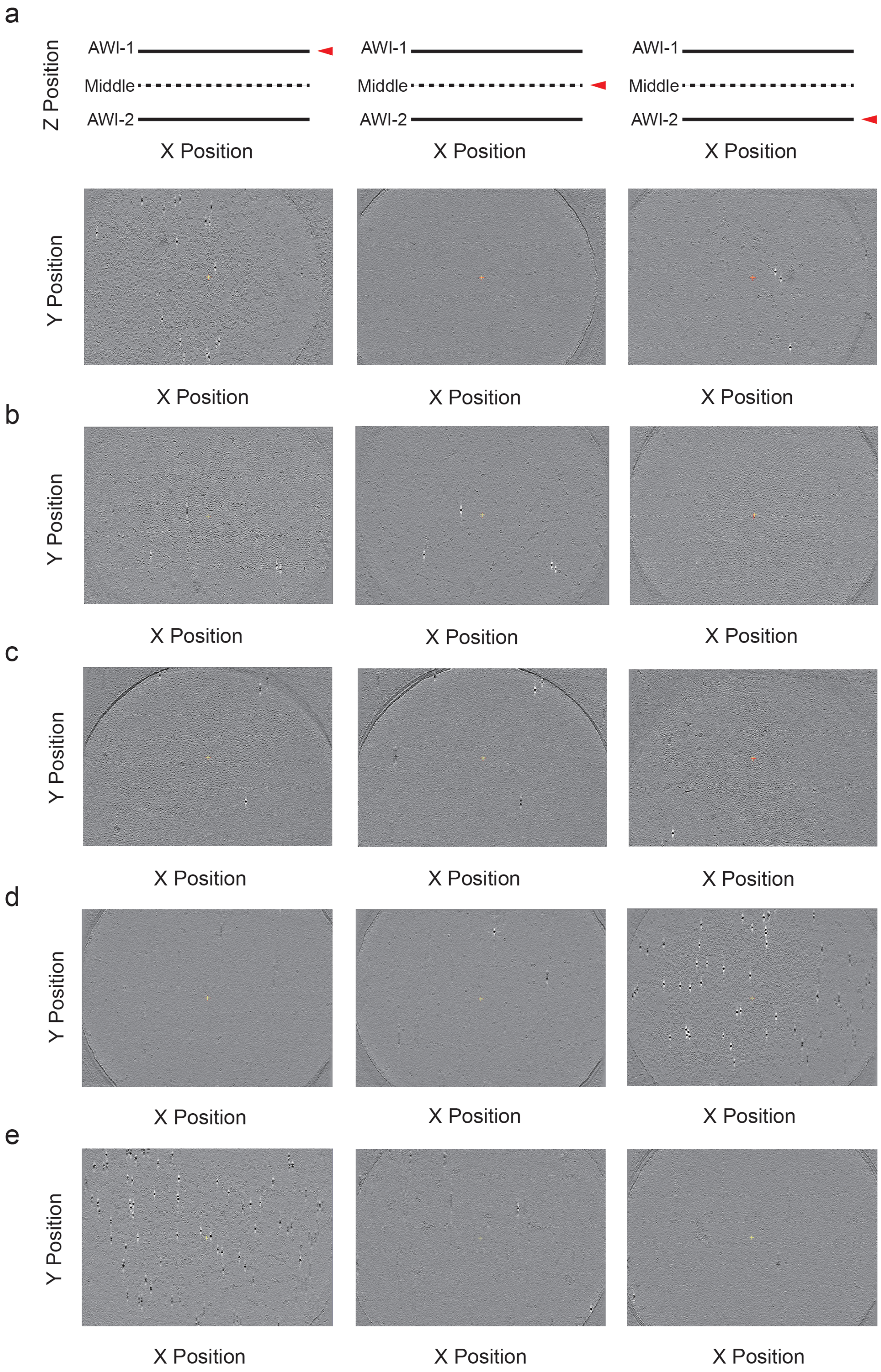

Supplementary Figure 4 Particle distribution of apoferritin in three different layers of tomograms. Comparison of images from upper air-water interface (left), inner (middle) and lower air-water interface (right) layers from tomograms for different datasets: apoferritin only **(a)**, apoferritin with MSBP **(b)**, MSBP only **(c)**, apoferritin with PEG **(d)**, apoferritin with $Pd(NO_3)_2$ **(e)**. The z position of each slice is indicated at the top.

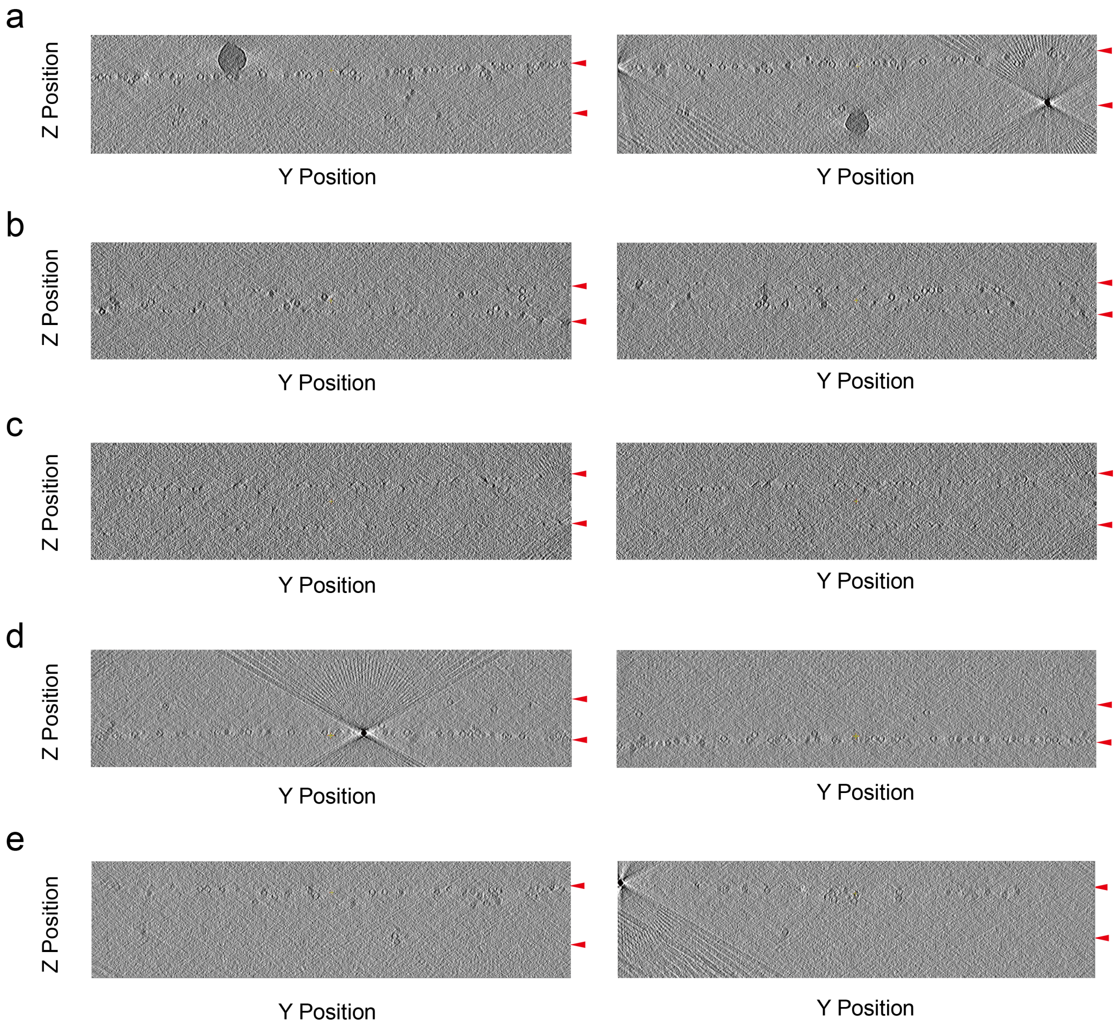

Supplementary Figure 5 Side view (Y-Z) segmentations of tomograms. Two different side view segmentations from tomograms for different datasets: apoferritin only **(a)**, apoferritin with MSBP **(b)**, MSBP only **(c)**, apoferritin with PEG**(d)**, apoferritin with $Pd(NO_3)_2$**(e)**. Red triangles refer to the air-water interfaces.

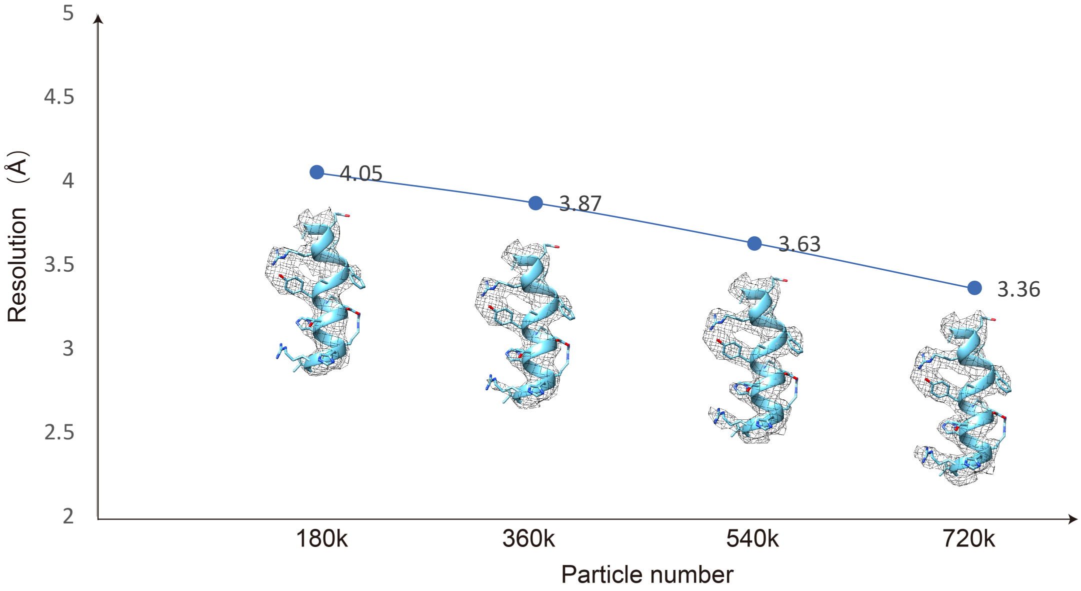

Supplementary Figure 6 The resolution of catalase monomer with MSBP is improved by increasing particle numbers. Representative cryo-EM densities of one catalase α helix fitted with the corresponding structural model (PDB:7P8W) are shown.

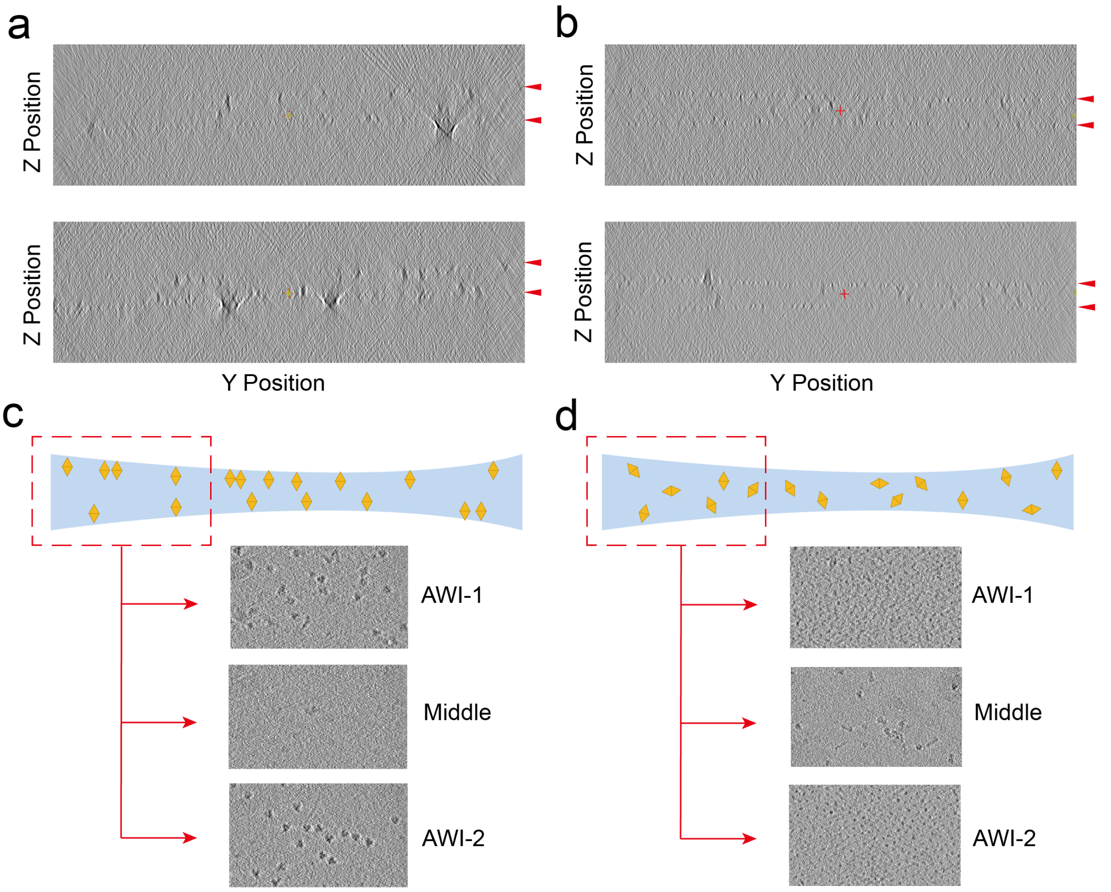

Supplementary Figure 7 Particle distribution of HA trimer in vitreous ice. Two different side view segmentations from tomograms of HA trimer without **(a)** or with **(b)** MSBP. **(c)** Schematic diagram shows particle distribution of HA trimer without MSBP in vitreous ice. Three layers along the Z axis of the tomogram indicated most of the particles are trapped in two AWIs, few particles are observed in the middle of the vitreous ice. **(d)** Schematic diagram shows particle distribution of HA trimer with MSBP in vitreous ice. Three layers along the Z axis of the tomogram indicated particles are observed in the middle of the vitreous ice.

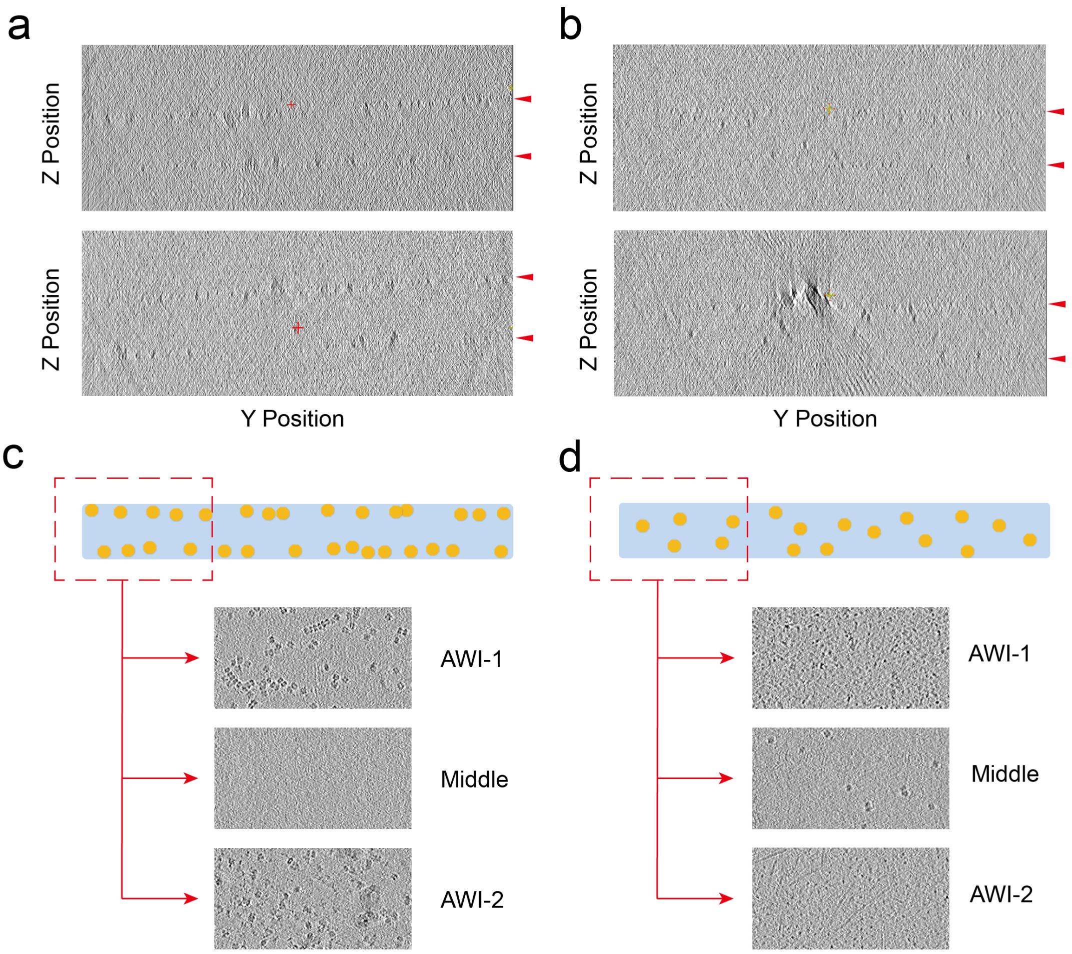

Supplementary Figure 8 Particle distribution of catalase in vitreous ice. Two different side view segmentations from tomograms of catalase without **(a)** or with **(b)** MSBP. **(c)** Schematic diagram shows particle distribution of catalase without MSBP in vitreous ice. Three layers along the Z axis of the tomogram indicated most of the particles are trapped in two AWIs, few particles are observed in the middle of the vitreous ice. **(d)** Schematic diagram shows particle distribution of catalase with MSBP in vitreous ice. Three layers along the Z axis of the tomogram indicated particles are observed in the middle of the vitreous ice.

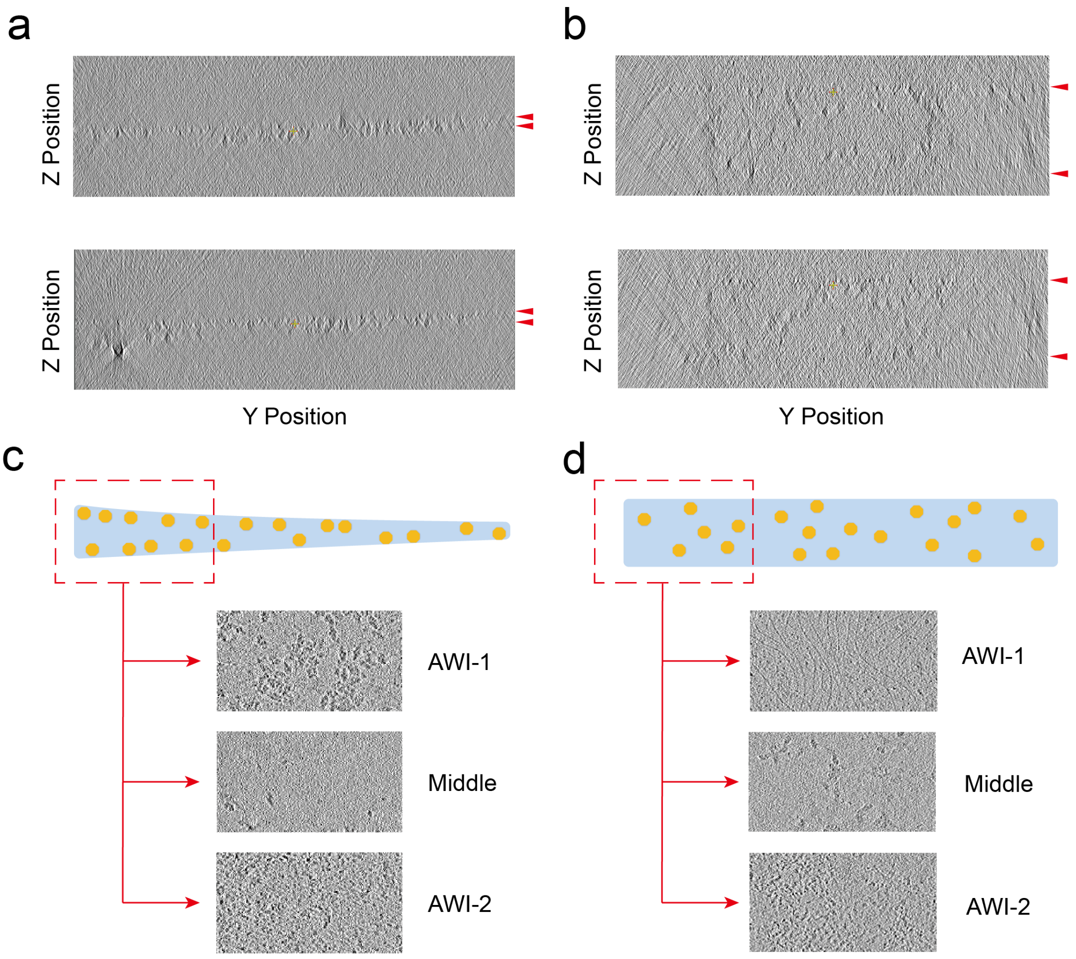

Supplementary Figure 9 Particle distribution of β-galactosidase in vitreous ice. Two different side view segmentations from tomograms of β-galactosidase without **(a)** or with **(b)** MSBP. **(c)** Schematic diagram shows particle distribution of β-galactosidase without MSBP in vitreous ice. Three layers along the Z axis of the tomogram indicated most of the particles are trapped in two AWIs, few particles are observed in the middle of the vitreous ice. **(d)** Schematic diagram shows particle distribution of β-galactosidase with MSBP in vitreous ice. Three layers along the Z axis of the tomogram indicated particles are observed in the middle of the vitreous ice.

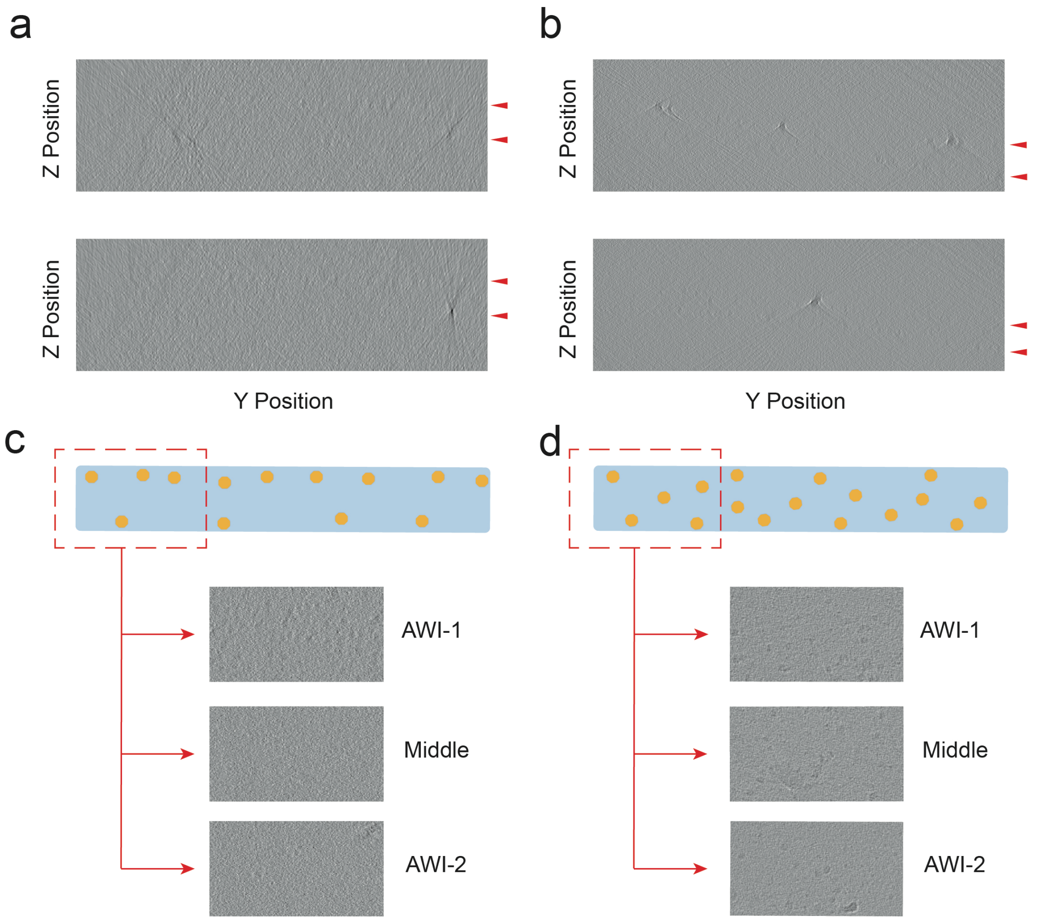

Supplementary Figure 10 Particle distribution of IMP1 in vitreous ice. Two different side view segmentations from tomograms of IMP1 without **(a)** or with **(b)** MSBP. **(c)** Schematic diagram shows particle distribution of IMP1 without MSBP in vitreous ice. Three layers along the Z axis of the tomogram indicated most of the particles are trapped in two AWIs, few particles are observed in the middle of the vitreous ice. **(d)** Schematic diagram shows particle distribution of IMP1 with MSBP in vitreous ice. Three layers along the Z axis of the tomogram indicated particles are observed in the middle of the vitreous ice.

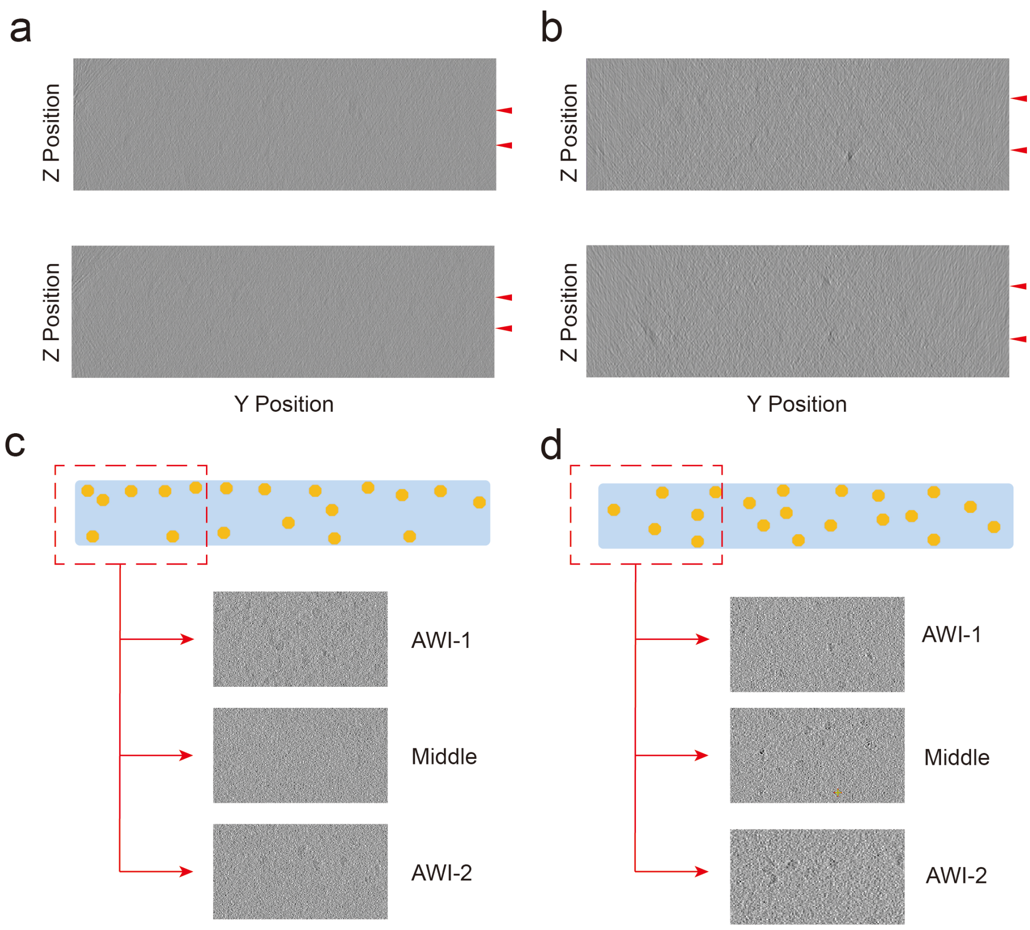

Supplementary Figure 11 Particle distribution of IMP2 in vitreous ice. Two different side view segmentations from tomograms of IMP2 without **(a)** or with **(b)** MSBP. **(c)** Schematic diagram shows particle distribution of IMP2 without MSBP in vitreous ice. Three layers along the Z axis of the tomogram indicated most of the particles are trapped in two AWIs, few particles are observed in the middle of the vitreous ice. **(d)** Schematic diagram shows particle distribution of IMP2 with MSBP in vitreous ice. Three layers along the Z axis of the tomogram indicated particles are observed in the middle of the vitreous ice.

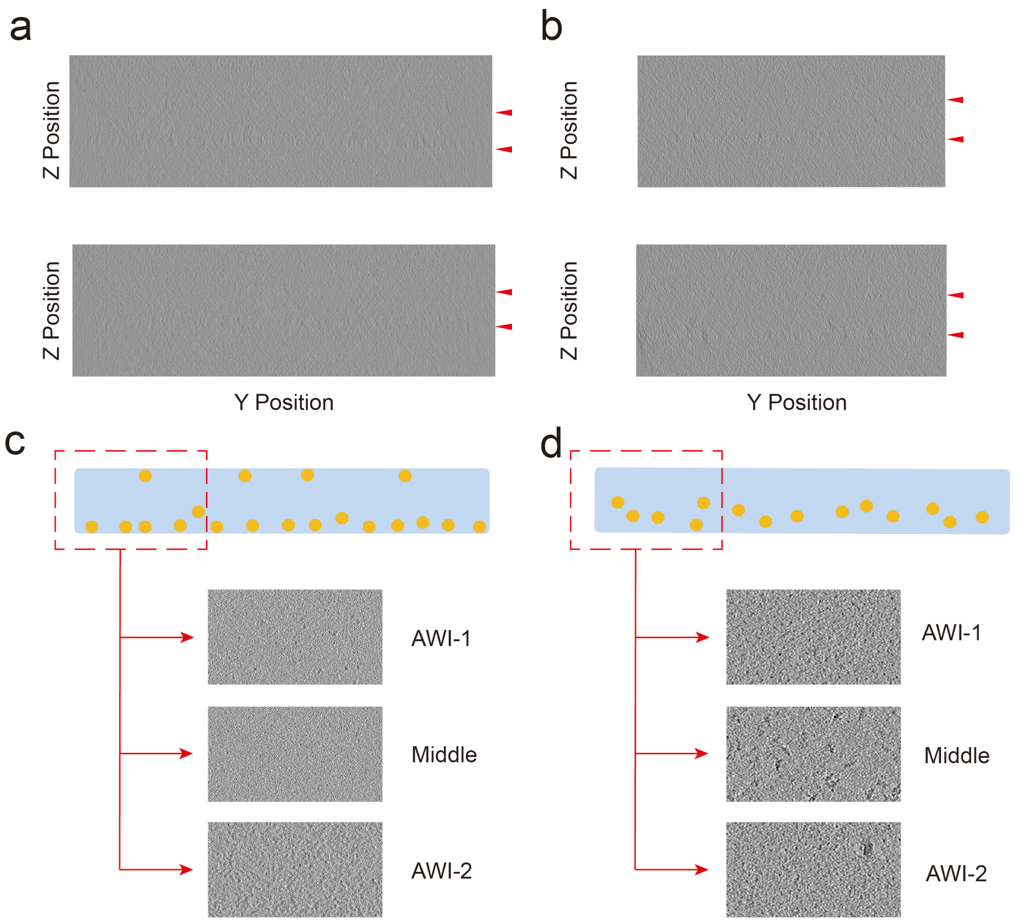

Supplementary Figure 12 Particle distribution of CSW complex in vitreous ice. Two different side view segmentations from tomograms of CSW complex without **(a)** or with **(b)** MSBP. **(c)** Schematic diagram shows particle distribution of CSW complex without MSBP in vitreous ice. Three layers along the Z axis of the tomogram indicated most of the particles are trapped in two AWIs, few particles are observed in the middle of the vitreous ice. **(d)** Schematic diagram shows particle distribution of CSW complex with MSBP in vitreous ice. Three layers along the Z axis of the tomogram indicated particles are observed in the middle of the vitreous ice.

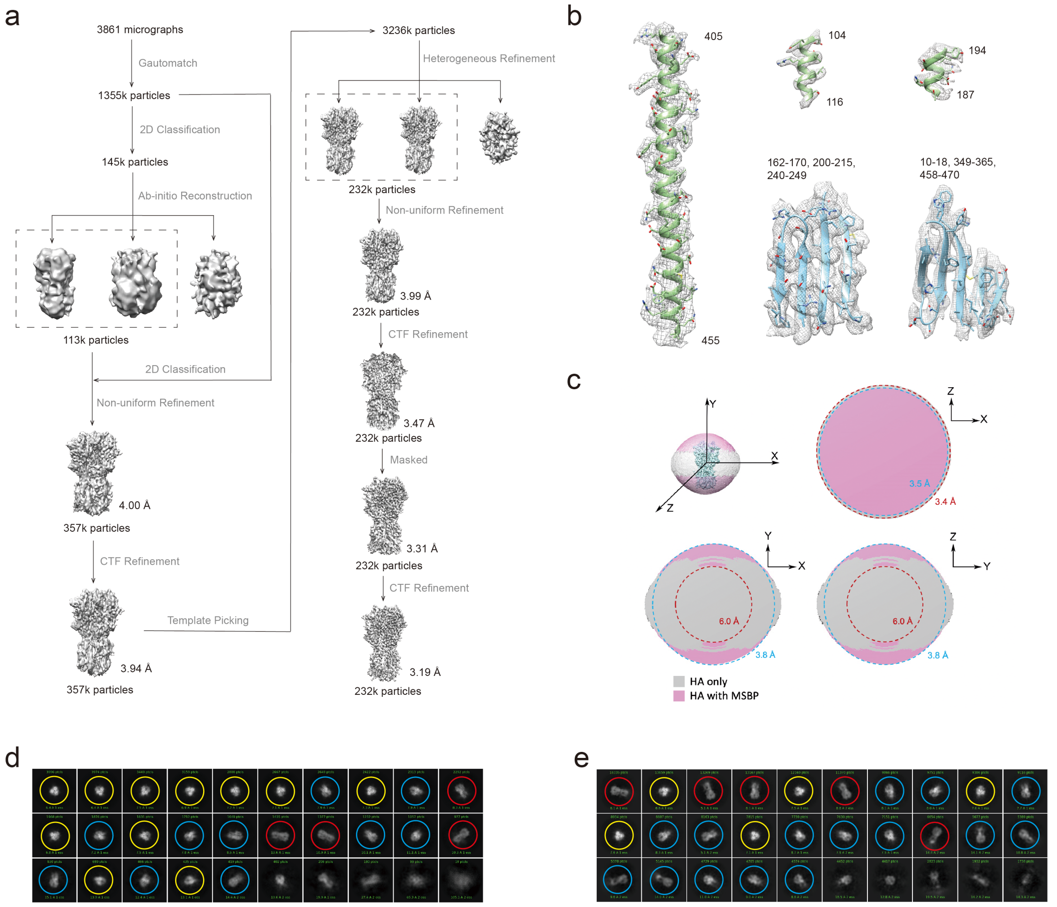

Supplementary Figure 13 Cryo-EM data analysis of hemagglutinin trimer. **(a)** Data processing workflow with the number of particles and the reconstruction resolution indicated at every step. **(b)** Representative cryo-EM densities of HA with MSBP. **(c)** Calculated resolution from different views for HA trimer without (grey) or with (purple) MSBP. **(d-e)** 2D averages of HA trimer without (d) or with (e) MSBP. Top views (yellow circles), side views (red circles) and tilted views (blue circles) are indicated.

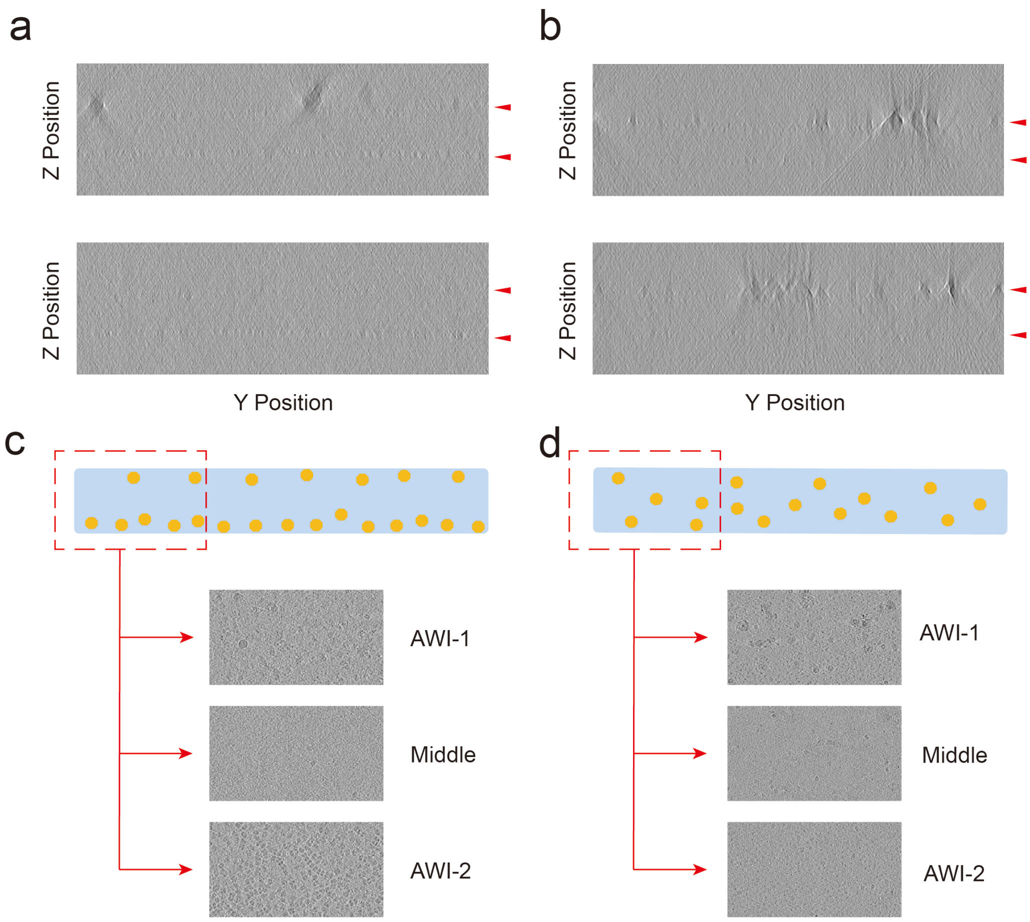

Supplementary Figure 14 Particle distribution of catalase under high concentration of salt in vitreous ice. Two different side view segmentations from tomograms of catalase prepared under high salt concentration (1M NaCl) without **(a)** or with **(b)** MSBP. **(c)** Schematic diagram shows particle distribution of catalase without MSBP in vitreous ice. Three layers along the Z axis of the tomogram indicated most of the particles are trapped in two AWIs, few particles are observed in the middle of the vitreous ice. **(d)** Schematic diagram shows particle distribution of catalase with MSBP in vitreous ice. Three layers along the Z axis of the tomogram indicated particles are observed in the middle of the vitreous ice.

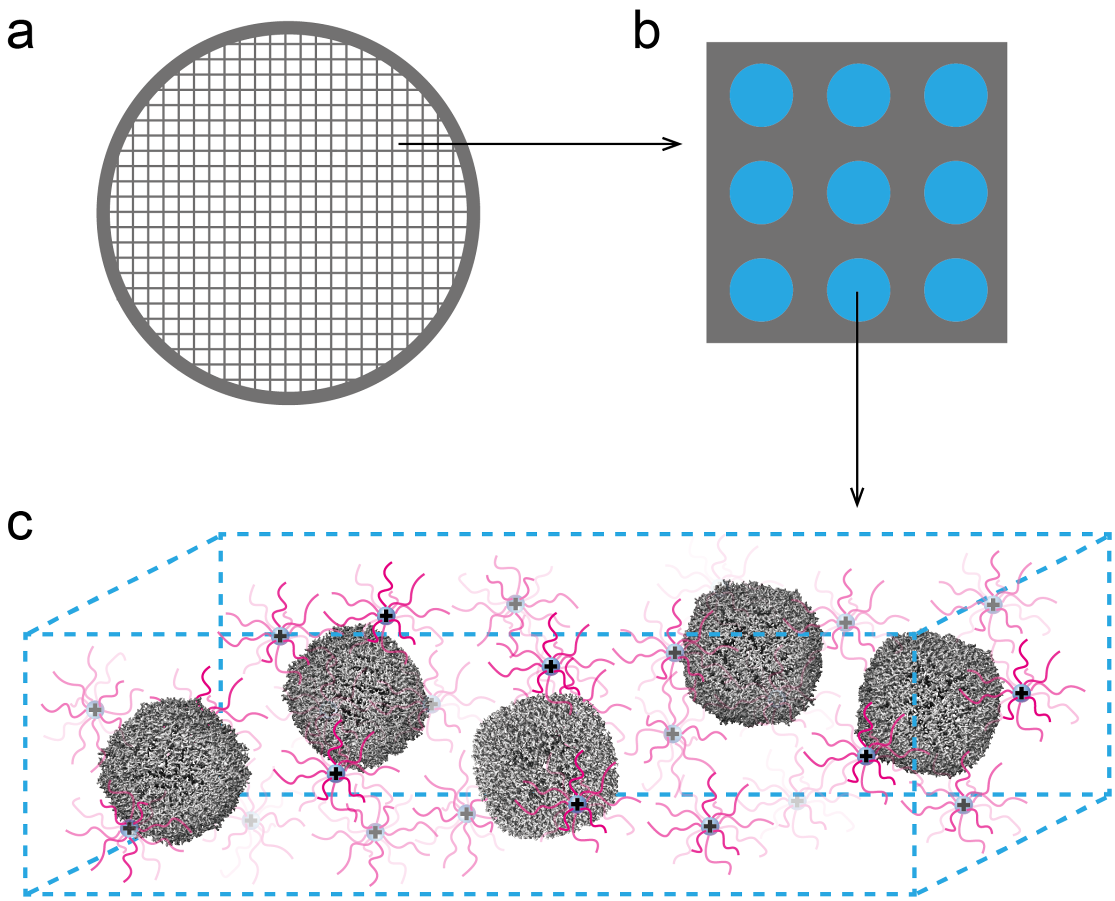

Supplementary Figure 15 Proposed model of MSBP interacting with protein particles. (a) A schematic of a typical holey carbon cryo-grid is shown. (**b**) A small region of cryo-grids with holes and carbon film is shown as blue and grey, respectively. **(c)** Proposed protein particles in vitreous ice with MSBP applied. Protein particles are denoted as grey sphere, the MSBP nanoclusters are indicated as pink PEG polymers connected to positively charged part.

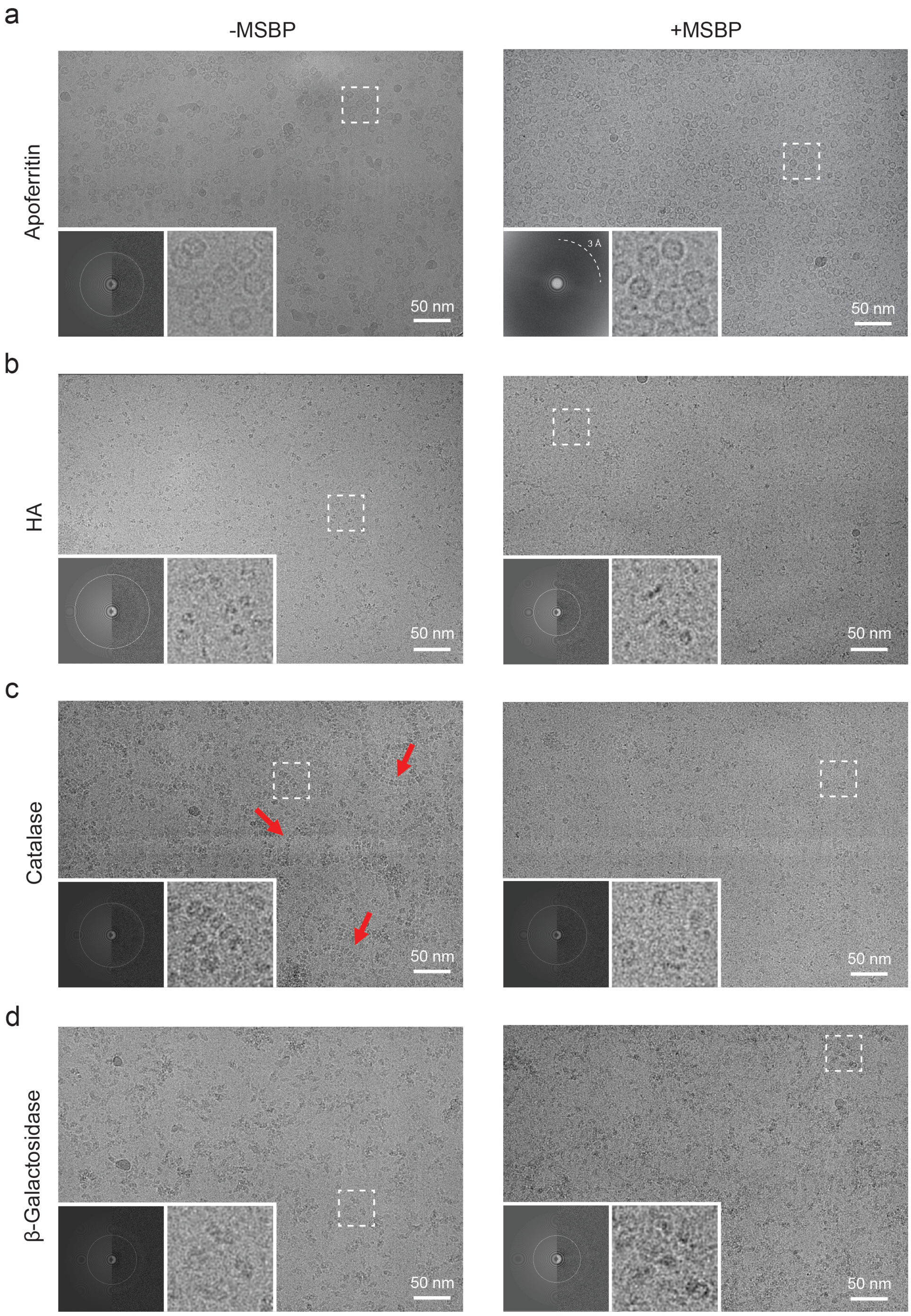

Supplementary Figure 16 MSBP does not affect particle picking from raw micrographs. (**a-d**) The representative raw micrographs of protein samples prepared without (left) or with MSBP (right) for apoferritin (a), HA (b), catalase (c), and β-galactosidase (d). The power spectrum and magnified view of region indicated as white dashed box are shown in the left corner of the corresponding micrograph. The filament-like structure formed by catalase under high concentration is indicated with red arrow.

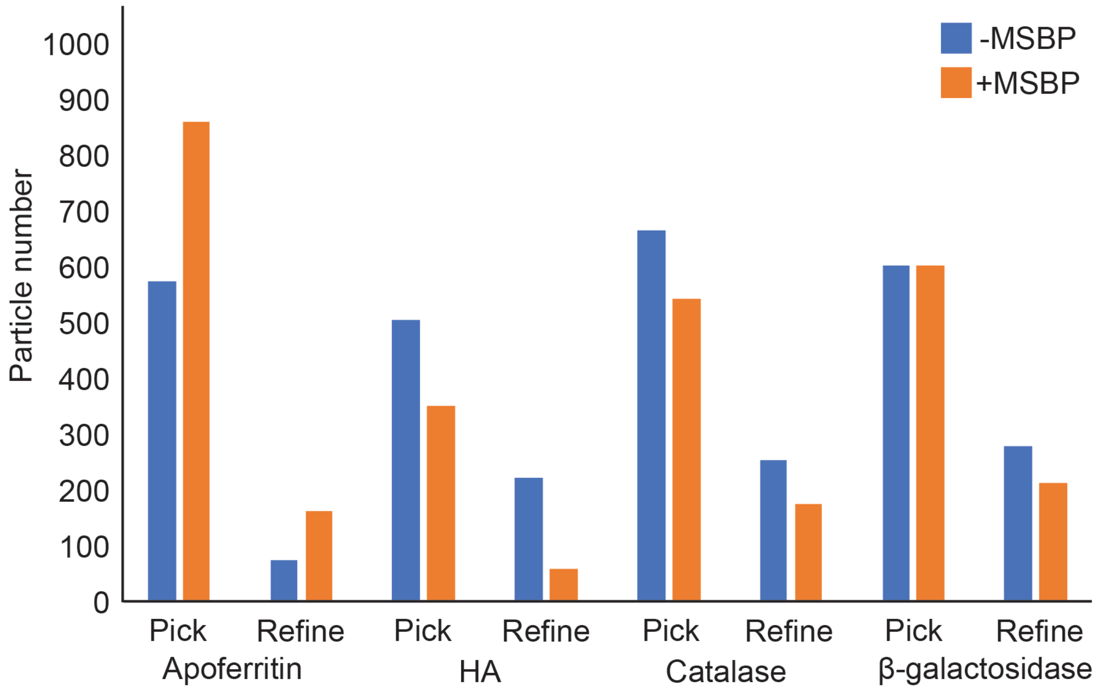

Supplementary Figure 17 Statistics of particle numbers per micrograph with and without MSBP. The distribution of particle numbers per micrograph is shown, with MSBP represented in orange and without MSBP represented in blue. The analysis includes apoferritin, HA-trimer, catalase, and β-galactosidase. For individual protein samples, the left side shows the Gautomatch picked particles, while the right side displays the particles used for the final reconstruction. The corresponding numbers of micrographs and particles for each sample are in the source data.

Supplementary Table 1 Summary of Cryo-EM Data Collection and Processing.

Protein	Apoferritin		Hemagglutinin		Catalase		β-Galactosidase	
MSBP	-	+	-	+	-	+	-	+
**Data collection**								
EM equipment	Titan Krios							
Voltage (kV)	300							
Detector	Gatan K3 summit							
Magnification	81000 x							
Pixel size (Å)	1.06							
Electron dose (e/Å²)	57.04	57.04	51.75	50.9	50.58	50.58	50.58	50.63
Defocus range (μm)	-1.3~-2.5	-1.3~-2.5	-1.3~-2.5	-1.3~-2.5	-1.0~-2.5	-1.0~-2.5	-1.0~-2.5	-1.0~-2.5
Collected movies	2,090	1,587	576	3,861	1,053	1,022	1,317	1,278
**Reconstruction**								
Software	cryoSPARC v2.15.0, RELION 3.1							
Final particles	150,000	255,785 /150,000	127,885	231,931	180,161	180,161	273,058	273,058
B-factors ($Å^2$)	-127.5	-87.9 /-88.4	-141.5	-149.5	-127.5	-129	-112.6	-131.6
Map resolution (Å)	2.53	2.16 /2.22	3.41	3.19	3.19	2.97	2.82	3.18
EMDB code		EMD-36313		EMD-36314		EMD-36315		EMD-36316
