# Supplementary material for: Metallo-supramolecular branched polymer protects particles from air-water interface in single-particle cryo-electron microscopy
Source: Commun Biol. 2024 Jan 9;7:65. doi: 10.1038/s42003-023-05752-8 (PMC10776832; doi:10.1038/s42003-023-05752-8)
Supplement: Supplementary file 3 — Description of Additional Supplementary Files [file 42003_2023_5752_MOESM3_ESM.pdf]

Description of Additional Supplementary Files

File name: Supplementary Data 1
Description: Source data related to Supplementary Figure 17.

File name: Supplementary Video 1
Description: Formation of MSBP in solution.

File name: Supplementary Video 2
Description: Reconstructed tomogram of apoferritin without MSBP.

File name: Supplementary Video 3
Description: Reconstructed tomogram of apoferritin with MSBP.

File name: Supplementary Video 4
Description: Reconstructed tomogram of MSBP only.

File name: Supplementary Video 5
Description: Reconstructed tomogram of proteins used in this study.
